# CT and cone-beam CT of ablative radiation therapy for pancreatic cancer with expert organ-at-risk contours

**DOI:** 10.1038/s41597-022-01758-9

**Published:** 2022-10-21

**Authors:** Jun Hong, Marsha Reyngold, Christopher Crane, John Cuaron, Carla Hajj, Justin Mann, Melissa Zinovoy, Ellen Yorke, Eve LoCastro, Aditya P. Apte, Gig Mageras

**Affiliations:** 1 Department of Medical Physics, Memorial Sloan Kettering Cancer Center, New York, NY 10065 USA; 2 Department of Radiation Oncology, Memorial Sloan Kettering Cancer Center, New York, NY 10065 USA

**Keywords:** Translational research, Scientific data, Computational science, Pancreatic cancer

## Abstract

We describe a dataset from patients who received ablative radiation therapy for locally advanced pancreatic cancer (LAPC), consisting of computed tomography (CT) and cone-beam CT (CBCT) images with physician-drawn organ-at-risk (OAR) contours. The image datasets (one CT for treatment planning and two CBCT scans at the time of treatment per patient) were collected from 40 patients. All scans were acquired with the patient in the treatment position and in a deep inspiration breath-hold state. Six radiation oncologists delineated the gastrointestinal OARs consisting of small bowel, stomach and duodenum, such that the same physician delineated all image sets belonging to the same patient. Two trained medical physicists further edited the contours to ensure adherence to delineation guidelines. The image and contour files are available in DICOM format and are publicly available from The Cancer Imaging Archive (10.7937/TCIA.ESHQ-4D90, Version 2). The dataset can serve as a criterion standard for evaluating the accuracy and reliability of deformable image registration and auto-segmentation algorithms, as well as a training set for deep-learning-based methods.

## Abstract (summary)

**Table:** 

Measurement(s)	Image Segmentation • Stomach • Duodenum • Small Intestine
Technology Type(s)	Computed Tomography • Kilovoltage Cone Beam Computed Tomography • Manual
Factor Type(s)	Treatment planning dose
Sample Characteristic - Organism	Homo sapiens

## Background & Summary

The dataset described in this article consists of CT and cone-beam CT (CBCT) images obtained from patients who received ablative hypofractionated radiation therapy for locally advanced pancreatic cancer (LAPC), i.e., cancer that has not yet spread to distant organs but cannot be removed completely with surgery. Cone-beam CT, typically acquired with an imaging system mounted on the treatment machine, is widely used as a means of volumetric (3-dimensional) image guidance for radiation therapy. It provides visualization of soft-tissue structures of interest with the patient in the treatment position, which enables target localization for patient positioning and assessment of changes in patient anatomy, such as changes in location and shape of nearby organs-at-risk (OARs). The latter is important for determining whether dose received by OARs (referred to as delivered dose) exceeds tolerances and modifications to the treatment plan are needed.

The need for daily volumetric image guidance is particularly acute for high-precision radiation treatments of LAPC. Pancreatic cancer has a poor outcome, with a 5-year survival rate of 11%^1^. Clinical and autopsy studies indicate that 30% or more of patients with LAPC die from complications related to local disease progression, i.e., continued proliferation and invasiveness of tumour cells within the pancreas^2^. Studies of conventionally fractionated radiotherapy (commonly 40–60 Gy in 1.8–2.0 Gy per fraction) have shown limited improvement in the survival duration of LAPC patients^2^. Hypofractionated (1–5 fractions) stereotactic body radiation therapy (SBRT) is capable of high-precision delivery of large doses to small tumour volumes. In treatment of tumours in lung or liver with SBRT, irradiation of small amounts of surrounding healthy tissue usually does not have substantial side effects; however, in LAPC the proximity of radiation-sensitive organs limits the dose that can be safely delivered to tumour (commonly 25–33 Gy in 3–5 fractions), thereby having little chance of improving long-term local tumour control or survival. Delivery of ablative treatment (i.e., providing sufficient dose to stop a tumour from growing and disrupt cellular and tissue function) for LAPC has shown encouraging outcome in local control and patient survival^3,4^. The treatment strategy involves delivery of a higher dose to a larger number of fractions (the earlier study prescribed 63–70 Gy in 28 fractions or 67.5 Gy in 15 fractions) than SBRT and abandons the traditional goal of uniform dose inside the tumour planning target volume (PTV), replacing it with a nonuniform dose that treats as much tumour as possible to high dose and delivers lower dose in areas abutting the highly sensitive and mobile gastrointestinal (GI) organs, specifically, the small bowel, stomach and duodenum. To maintain tolerance, an accurate assessment of organ-at-risk (OAR) position relative to the high dose region is vital. Techniques to ensure safe dose delivery include advanced organ motion management and image guidance during treatment. Daily breath-hold cone-beam CT (CBCT) scans are acquired in the treatment room before the patient receives treatment, and current clinical practice involves visual inspection of OAR displacement and deformation between the planning CT and CBCT to assess the need for treatment plan modification. The visual inspection process requires clinical experience and is time consuming, which limits more widespread use of this treatment strategy. In addition, computation of delivered dose to OARs is desirable for quantitative assessment.

For these reasons, accurate CT-to-CBCT deformable registration and automatic segmentation tools for guiding radiation treatment are desirable. CBCT image quality in pancreas poses challenges, however, which adversely affect the accuracy and reliability of current commercially available image registration tools. CBCT images in this disease site exhibit low soft-tissue contrast and image quality is affected by artefacts, which include a large component of x-ray scattering in the patient, residual motion-induced blurring and streaking, and cupping artefacts in the limited-view CBCT scans (described later). Therefore, there is a need for such datasets to develop and test new image registration and segmentation methods.

The dataset reported here possesses several characteristics that make it unique, to our knowledge, among publicly available archived datasets. It consists of planning CT and CBCT scans obtained from patients with LAPC who received ablative hypofractionated radiation treatments^4^. All image acquisitions and treatment were carried out with the patient in a deep-inspiration breath-hold state to increase the visibility of target tissues and OARs and to reduce motion-induced distortions, thereby increasing segmentation accuracy and consistency. The dataset includes expert-drawn gastrointestinal OAR segmentations on the plan CT and CBCT images, thus providing a criterion standard for investigating CT-to-CBCT registration and automatic segmentation methods for this disease site.

This dataset was used in developing and evaluating a deep-learning-based deformable registration method to predict OAR segmentations on the CBCT derived from segmentations on the planning CT^5^. Deep-learning-based methods of deformable registration and automatic segmentation recently have shown promise in addressing the challenges with medical images^6^, but depend on the availability of such images for training. To date, there has been a lack of publicly accessible radiotherapy image data in abdominal sites that are suitable for this purpose. Our findings in the previous study have indicated the utility of this dataset for training deep-learning-based CT-to-CBCT deformable registration and OAR segmentation in the pancreatic disease site, and we expect it to be useful for training and evaluating other deep-learning-based methods.

## Methods

### Data collection

The dataset was collected and prepared under an IRB-approved retrospective research protocol (IRB #18–227) at Memorial Sloan Kettering Cancer Center (MSKCC). The aims of the protocol were to develop a new deformable CT-to-CBCT registration algorithm for determining delivered dose to organs-at-risk in the CBCT images, and to evaluate its accuracy in retrospective analysis of patient data. Han *et al*. have reported on a study stemming from this protocol and cited the data described here^5^. The images were selected from 40 patients (one treatment planning CT and two CBCT scans from each patient) with LAPC who underwent ablative hypofractionated radiation therapy at MSKCC between years 2016 and 2018.

**Fig. 1:**
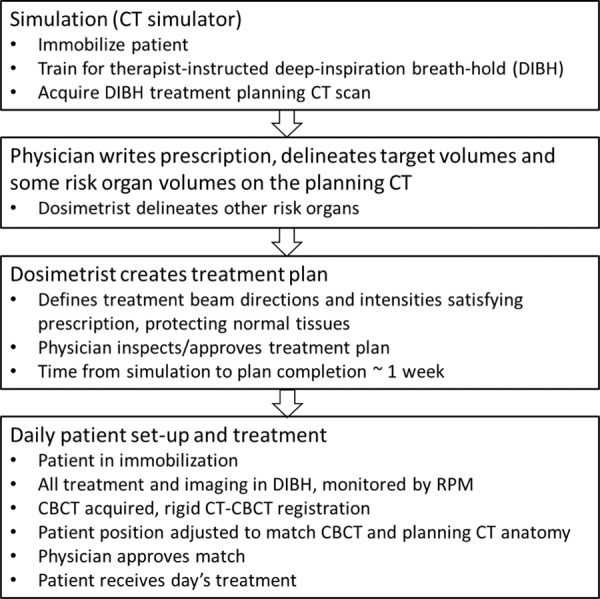
Flowchart of the treatment process of patients in this dataset.

We describe our clinical process. Each patient was treated with ablative radiation therapy delivered with 6 megavolt (MV) x-rays on a medical linear accelerator (TrueBeam v. 2.5, Varian Medical Systems) for pancreatic cancer following the regimen described by Reyngold *et al.*^4^. In the clinical workflow (Fig. 1), the physician prescribes the dose to the target volume and the total number of treatment fractions (in this group, 15 or 25 fractions delivered one per day, 5 days/week). The target volume includes overt tumour which is prescribed the highest – the ablative – radiation dose, tissue at risk for harbouring tumour, which receives an intermediate dose and tissue planned to receive a lower but still significant dose because it may contain even smaller concentrations of tumour, especially if anatomy at treatment changes slightly. Normal organs in proximity to the pancreas, such as the stomach, duodenum and bowel, are very sensitive to radiation so a key treatment requirement is to protect these risk organs and prevent complications.

Preparation for treatment begins with a simulation session at which the patient is positioned supine within a custom-fabricated immobilization device, with arms extended above the head, and given small tattoos such that the patient’s position is reproductible at each treatment session. A diagnostic quality CT scan, referred to as the planning CT scan, is acquired at the simulator, which is a specially equipped CT-scanner with a flat couch that reproduces the configuration at treatment (Brilliance Big Bore, Philips Health Systems; or Discovery ST, GE Healthcare). Iodinated contrast is used during the scan; the acquisition settings were acquisition mode = helical, tube voltage = 120 kVp or 140 kVp, slice thickness = 1.5–3.0 mm, reconstruction diameter = 500–700 mm, matrix size = 512 × 512, pixel size = 0.98–1.37 mm (dependent on reconstruction diameter).

Because the pancreas and the risk organs can move by 1 cm or more with normal respiration, in our clinical practice, whenever possible, patients receiving ablative pancreatic cancer treatment are simulated and treated in a coached deep inspiratory breath-hold state (DIBH), so that the anatomy at each treatment session reproduces simulation as closely as possible. Specifically, the simulating therapist trains the patient to perform the breath-hold and then acquires a breath-hold-monitored planning CT-scan to be used as the anatomical basis of the patient’s radiation therapy treatment plan and as the standard by which the patient is set up at each treatment. Breath-hold monitoring at simulation (Real-Time Position Management, Varian Medical Systems) and treatment (Respiratory Motion Management, TrueBeam, Varian Medical Systems) is performed with a respiratory monitor, which tracks the vertical motion of an infrared reflective marker that is taped to the patient’s chest and displays a graph of this motion. For normal breathing, the graph is oscillatory but during a good breath-hold, it differs from a horizontal line within user-set tolerances (referred to as the gate), usually 3–4 mm^7^. The simulating therapist acquires the planning CT scan when the respiratory monitor shows a sufficiently flat breath-hold.

The physician delineates the relevant tumour and risk organ anatomy on the planning CT scan. Then a dosimetrist generates the radiation therapy treatment plan with a commercial planning system (Eclipse v. 15.5, Varian Medical Systems). The treatment plan specifies the radiation beam sizes and directions to be used at the treatment machine and, based on beam data measured by the department’s physicists, predicts the radiation doses to be delivered to the tumour, to each delineated risk organ and to intervening tissue. Each treatment plan is customized to the individual patient’s anatomy as captured on the planning CT. The treatment plan is reviewed by the physician; it may be modified several times before it is approved, and the patient can proceed to treatment. Further description of the general treatment planning process can be found elsewhere^8^.

At each treatment, the patient is positioned in the immobilization device on the treatment machine couch and respiration is monitored. All imaging and treatment are done in the DIBH state, coached by the treating therapist. Before beginning treatment, a kilovoltage CBCT scan is acquired using the treatment machine imaging hardware and reconstruction software. Of the 80 CBCT scans in this dataset, 58 were limited-view scans acquired with a 200-degree gantry rotation with no detector offset, and the remaining scans (referred to as full-view scans) with 360-degree gantry rotation and lateral detector offset. The acquisition time of the limited-view CBCT scans was shorter (approx. 45 s vs 60 s for full-view scans). The reconstruction dimensions for limited-view scans were 25 cm in diameter and 18 cm in length, whereas for full-view scans they were 46.5 cm and 15.7 cm, respectively. Acquisition parameters were: scan option = smooth, X-ray tube current = 20–100 mA, tube voltage = 125.0 kVp, exposure = 270–756 mA, Exposure time = 700 s, source-detector distance 150 cm, source-isocentre distance = 100 cm, field of view = 250 × 250 mm^2^, matrix size = 512 × 512, pixel spacing = 0.5 mm (limited-view) or 0.9 mm (full-view), slice thickness = 2.0 mm or 3.0 mm. The article by Srinivasan *et al*. reviews applications of cone-beam CT to radiation therapy^9^.

Using the treatment machine rigid image registration capability, the treating therapist adjusts the patient’s position determined in the CBCT to best replicate the anatomy of the planning scan, by aligning to an implanted fiducial marker near the tumour, or to a stent implanted during surgery. Attention is also given to the position of the ablative part of the target relative to the risk organs. At least one CBCT scan is acquired at each treatment and the therapist’s adjustments must be approved by an attending physician. During treatment, the respiratory gating (RPM) system allows the treatment beam to be delivered only if the breath-hold is within the gate. The article by Brock reviews image registration in radiation therapy^10^.

**Fig. 2:**
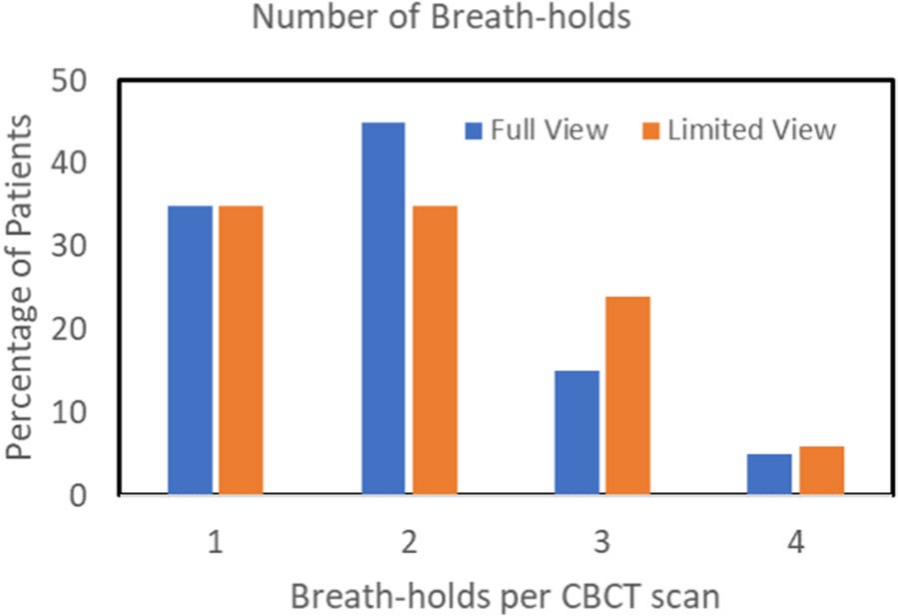
Percentage of patients versus estimated number of breath-holds per CBCT scan.

We briefly describe how a CBCT scan was acquired with patient breath-hold. The breathing trace from the respiratory monitor was displayed on the treatment machine console during treatment. For the patients in this cohort (years 2016–18), the therapists watched the trace and manually enabled gantry rotation with CBCT acquisition when the breath-hold level was within the approved gate (3 or 4 mm wide) and paused acquisition and gantry rotation otherwise. The patients received at least one CBCT scan just before each treatment. Most received at least one full-view scan on the first treatment day. On later treatment days, some patients received limited-view scans but, according to physician preference, some received full-view scans on all treatment days. Figure 2 shows the percentage of patients vs estimated number of breath-holds during CBCT acquisition. The treatment machine system version at that time did not record the breathing trace of the CBCT scans after treatment was completed, so examination of retrospective data did not provide a direct check of the number of breath-holds during CBCT. For some patients, however, the breathing traces during delivery of each treatment beam were recorded together with the number of times that the treatment beam was enabled. We therefore estimated the number of breath-holds per CBCT by assuming that a patient’s ability to perform DIBH on a given day was similar for the CBCT and the treatment fields. We used the first 60 s of the first treatment field for 20 patients on a day when they received full-view CBCT scans, and the first 45 s from 17 patients when they received limited-view CBCT. For both full-view and limited-view scans, the median number of breath-holds estimated in this way was 2 and the range was 1–4; the distribution of the number of breath-holds was quite similar.

### Data review and processing

**Fig. 3:**
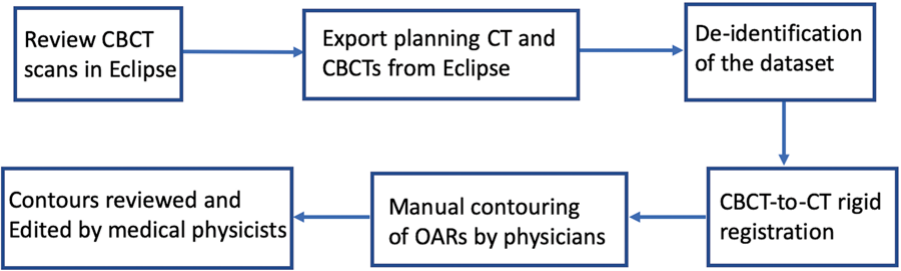
Flowchart of the review and processing of the dataset.

**Fig. 4:**
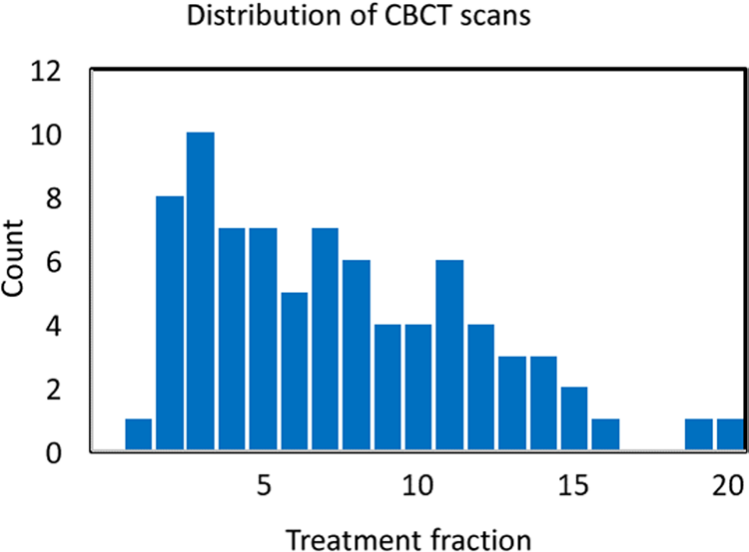
Histogram of CBCT scans by treatment fraction.

A flowchart of data review and processing is shown in Fig. 3. Review of CBCT images for inclusion in the protocol was performed with a commercial system (Offline Review, ARIA Oncology Information System for Radiation Oncology v. 15.5, Varian Medical Systems). Selection was based on image quality, specifically, on the visibility of OAR boundaries, to facilitate subsequent manual segmentation. Figure 4 shows the distribution of treatment fractions from which CBCT scans were chosen for this dataset. The majority of scans were from the first seven fractions. There were two reasons for this. First, patients generally exhibited more gas in the GI tract later in treatment, earlier scans had less gas and the image quality usually was better; thus, the organ delineations provided by the radiation oncologists was subject to less uncertainty. Second, in the context of clinical application, it is desirable to detect deviations in OAR positions relative to the planning CT at the earliest onset, so as to adjust the treatment plan promptly if needed.

Following image review, one planning CT and two CBCT scans (DICOM image and RT Structure Set) selected from the patient’s treatment plan were exported (Eclipse Export, ARIA) to a secure server. An in-house de-identification program (tested and certified by MSKCC Information Security Office) was used to remove patient health information (PHI) from the dataset. The anonymized datasets were imported to a commercial software system for image registration and segmentation (MIM v. 6.9.7, MIM Software Inc.). CBCT-to-CT rigid registration was applied that replicated actual treatment, i.e., by aligning to an implanted fiducial marker or stent, followed by resampling of the CBCT to the same voxel size and FOV as the planning CT (henceforth referred to as resampled CBCT). In cases where there were multiple markers, the one closest to the plan isocentre was chosen for alignment. A volume of interest (VOI) was defined on the image scans by adding a 1 cm margin (3-D expansion) to the low-dose PTV (i.e. the volume to receive 37.5 Gy or 45 Gy in 15 or 25-fraction treatment plans, respectively^4^). The size of the VOI was chosen as such that the portions of OARs receiving moderate to high dose were included while excluding the periphery of the CBCT volume, which was subject to cupping artefacts.

**Fig. 5:**
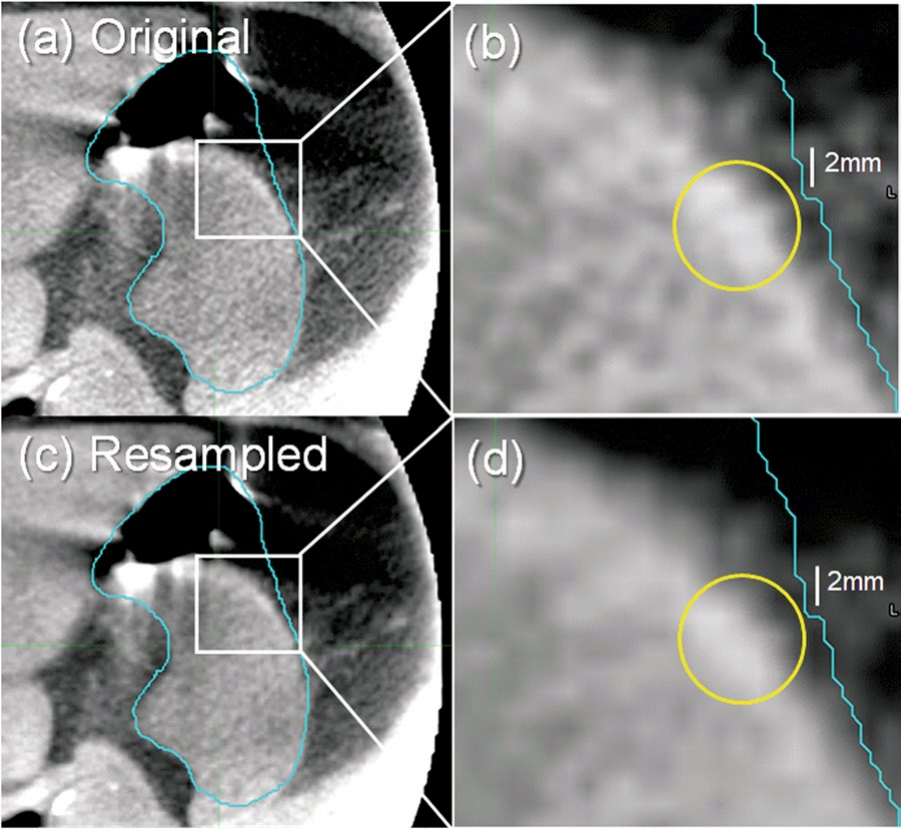
Example of a limited-view CBCT axial slice (**a**) in original image format, (**b**) magnified portion of original image, (**c**) resampled image, and (**d**) magnified portion of resampled image. Stomach is outlined in all panels. Circles in panels (**b**) and (**d**) indicate areas where pixel-scale artifacts are visible.

We examined whether resampling of the CBCT introduced image artifacts, owing to the large in-slice downsampling, i.e., increase in pixel size from 0.5 mm in the limited-view CBCT to 0.98 or 1.37 mm in the planning CT. Figure 5 compares axial slices from the original limited-view CBCT (top left panel) and resampled CBCT (bottom left). The small granular texture within the stomach (outlined) and surrounding tissues of the original image is more blurred in the resampled image; however, the sharpness of organ boundaries is well preserved. Examination of a magnified view of a portion of the stomach boundary (right panels) reveals faint pixel-scale artifacts in both images (circles) which are slightly more pronounced in the resampled image but have minimal effect on the visibility of the organ boundary. In the application of the resampled CBCT images to our prior study^5^, images were smoothed by a 3D Gaussian kernel with a standard deviation of 2 mm in each of the 3 principal directions, prior to training and evaluation of the deep-learning-based method, thereby further reducing pixel-scale artifacts. Further, the availability of the CBCT images in their original format in this dataset allows researchers to investigate their own resampling methods.

**Fig. 6:**
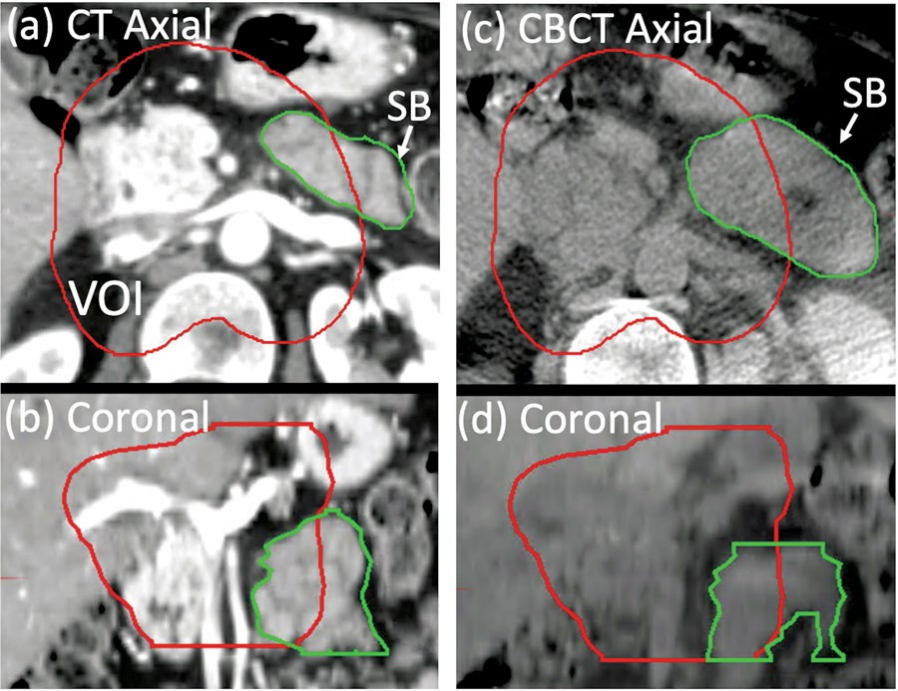
Example of physician-drawn small bowel (SB) contours on (**a**) axial and (**b**) coronal views of the planning CT, and on corresponding views (**c**), (**d**) of the CBCT scan. The volume of interest (VOI) is also outlined.

**Fig. 7:**
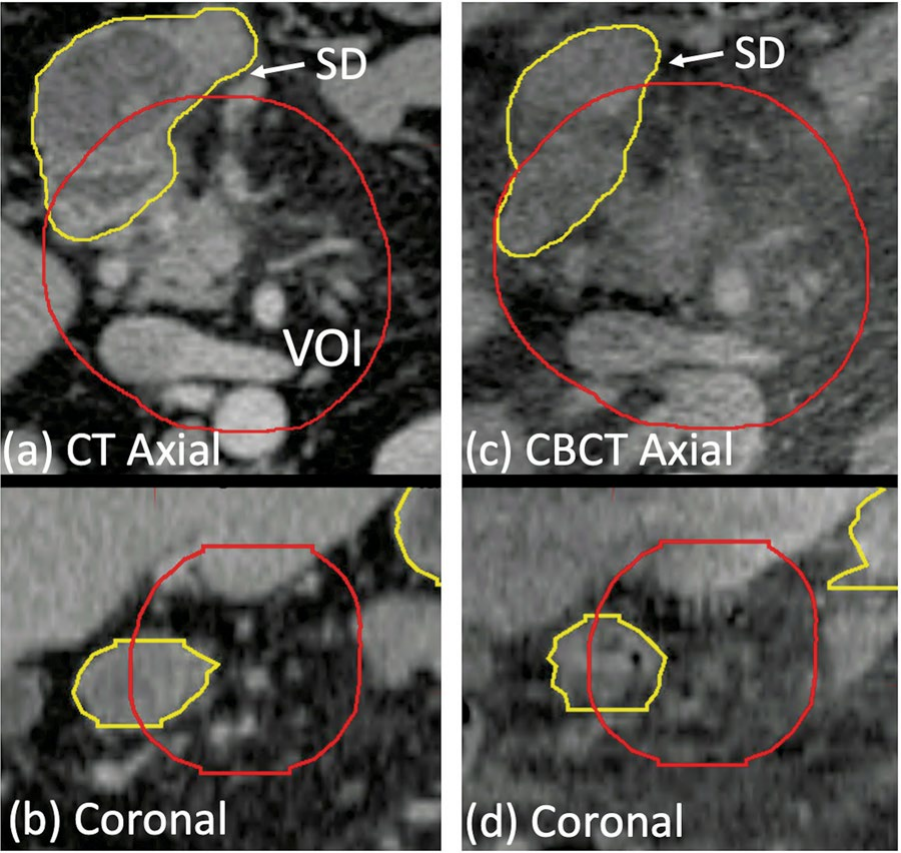
Example of physician-drawn stomach/duodenum (SD) contours on (**a**) axial and (**b**) coronal views of the planning CT, and on corresponding views (**c**), (**d**) of the CBCT scan. The volume of interest (VOI) is also outlined.

The motivation of this data set, and the associated study^5^, was to develop a tool for quantitatively assessing dose to OARs in ablative radiation treatment of LAPC. As mentioned above, the challenge in this type of treatment is the delivery of ablative tumoricidal doses near highly sensitive gastrointestinal OARs, consisting of (1) stomach and duodenum, and (2) the rest of the small bowel. The manual segmentations of this dataset therefore focused on these OARs in accordance with the toxicity reduction priorities of the clinical protocol^4^. The patient image sets were distributed among six experienced radiation oncologists for manual segmentation of the OARs, such that the same physician delineated all three image sets belonging to the same patient. The stomach and duodenum contours included stomach from the gastroesophageal junction through the pylorus as well as the first two segments of the duodenum. The small bowel contours started at the third segment of the duodenum. On the CBCT scans, only the portions of OARs within the VOI were delineated, whereas on the planning CT scans, the delineations extended to 2 cm outside of the VOI (in some cases contours may extend beyond these limits). Following OAR delineation by the physicians, two trained medical physicists (JH and GM) reviewed and further edited the contours, to ensure adherence to delineation guidelines. Examples of manual segmentations of small bowel and stomach/duodenum are shown in Figs. 6 and 7, respectively. The median volume of small bowel drawn on CBCT scans inside VOI is 34.8 cm^3^ (range: 4.0–113.4 cm^3^); the median volume of stomach and duodenum is 78.5 cm^3^ (range: 17.4–221.8 cm^3^). The median volume of small bowel drawn in planning CT scans inside VOI is 38.5 cm^3^ (range: 3.9–124.2 cm^3^) and the median volume of stomach and duodenum is 68.4 cm^3^ (range: 16.0–132.8 cm^3^).

In addition, lung volumes were delineated on the planning CT, using the Region Grow tool with lung settings, available in MIM. The delineations were transferred to the associated CBCT and manually aligned to the portion of the lungs visible in the images. The lung segmentations served to further process the images in the prior study^5^, specifically, to define regions to be excluded from image processing to fill gas pockets. Although this processing was not included in the current dataset, users may find the lung contours useful for applying their own gas-pocket filling algorithms.

## Data Records

The dataset^11^ is available from The Cancer Imaging Archive (TCIA, https://www.cancerimagingarchive.net/). In Version 2, there are 40 patient datasets, each consisting of one planning CT and four CBCT image sets (two in original image format and two in resampled format), associated radiation therapy (RT) structure sets and RT dose sets (total of 200 image sets, 130 RT structure set files and 40 RT dose files) (Table 1). All image, structure set and dose files conform to the DICOM standard for radiotherapy data, such that image files follow the Computed Tomography IOD (Image Object Definition, a DICOM term), structure set files follow the RT Structure Set IOD, and dose files follow the RT Dose IOD. IOD details are described in vendor-provided DICOM conformance statements^12,13^. The storage requirement for the dataset is 13.3 GB.

**Table 1:** Dataset Version 2 summary.

Study subjects	Patients receiving ablative radiation therapy for locally advanced pancreatic cancer
Modalities	Computed Tomography (CT)
	Radiation Therapy (RT) Dose
	RT Structure Set
Number of patients	40
Number of image sets per patient	5 (1 planning CT; 2 resampled CBCT; 2 original CBCT)
Number of RT structure set files per patient	In 30 patients:
	• 3 (1 planning CT; 2 resampled CBCT)
	In 10 patients:
	• 4 (1 planning CT; 2 resampled CBCT; 1 original CBCT)
Number of RT Dose files per patient	1 (planning CT)
Total number of image sets	200
Total number of RT Structure Set files	130
Total number of RT Dose files	40
Manually segmented organs	Small bowel; stomach/duodenum

For the purposes of meeting TCIA’s requirements for de-identification, the segmentations that were manually drawn on the de-identified images (described in the Methods section) were transferred to the original image data using image registration tools in MIM. The original image data and transferred segmentations were then processed using NIH-approved de-identification software provided by TCIA prior to submitting the dataset to the TCIA website. The RT structure file of the planning CT contains five structures: small bowel, stomach/duodenum, left lung, right lung and VOI (described in the Acquisition section), which are named as Bowel_sm_planCT, Stomach_duo_planCT, LUNG_L, LUNG_R, and ROI (identical to VOI). The RT structure file of each resampled CBCT scan contains four structures: small bowel, stomach/duodenum, left lung and right lung, which are named as Bowel_sm_CBCT, Stomach_duo_CBCT, LUNG_L, and LUNG_R.

Each planning CT is accompanied with a DICOM RT Dose file, consisting of the dose distribution calculated on the planning CT and which enables dosimetric analysis of the structures. Since the resampled CBCT scans have been registered to the planning CT via fiducial alignment, dosimetric analysis is also possible for structures defined in the resampled CBCT scans. Each resampled CBCT has a corresponding CBCT in the original image format. Since it is these images that are available in a clinical environment, they are useful for developing algorithms to be applied in the clinic. Ten of these original-format CBCT scans, from ten different patients (patient IDs ending in 003, 006, 012, 013, 015, 021, 025, 030, 035 and 036), are each accompanied by a DICOM RT Structure Set file containing repeat manual segmentations of the OARS by two independent observers and contained within the ROI volume. There are five structures in each file: BowelSmObs1, BowelSmObs2, ROI, StomachDuoObs1 and StomachDuoObs2. Here, label BowelSm refers to small bowel, StomachDuo to stomach/duodenum, Obs1 and Obs2 to Observers 1 and 2 respectively, where BowelSmObs1 and StomachDuoObs1 correspond to the segmentations in the resampled CBCT. The contours from Observer 1 are also the reference (approximate ground truth) segmentations used in the technical validation (see next section). In some cases, the ROI has been enlarged, relative to that in the resampled CBCT, so as to include sufficient volume (at least 10 cm^3^) of the repeat segmentations in order to obtain meaningful validation measurements. The repeat segmentations are included in this dataset as an informative way of seeing where the two experts agree and disagree in the CBCT images.

When data of each subject is downloaded from TCIA using NBIA Data Retriever, the images are stored in the path: ..\Pancreatic-CT-CBCT-SEG\ PatientID\StudyDate-NA-PANCREAS- StudyInstanceUID(last 5 digits)\SeriesNumber.000000-SeriesDiscription-SeriesInstanceUID(last 5 digits)\1-xxx.dcm where xxx starts from 001 to ###, where ### is the total number of image slices in the scan. The associated RTSTRUCT DICOM file can be found in the same path:..\Pancreatic-CT-CBCT-SEG\ PatientID\StudyDate-NA-PANCREAS- StudyInstanceUID(last 5 digits)\SeriesNumber.000000-SeriesDiscription-SeriesInstanceUID(last 5 digits)\1-1.dcm, with different SeriesInstanceUID.

## Technical Validation

The interobserver variation of the manual segmentations on the CBCT was analysed by calculating Dice similarity coefficient (DSC) between OAR segmentations drawn by two independent observers on a subset of ten CBCT images, each from a different patient, and of sufficient image quality that the OAR boundaries were deemed to be visible within the VOI. The planning CT and associated OAR segmentations were available to the observers as a guide, but they were blinded to any prior segmentations on the CBCT. The mean ± one-standard-deviation of DSC for small bowel (n = 10) was 0.82 ± 0.07 (range 0.69–0.90); for stomach/duodenum (n = 10) it was 0.86 ± 0.06 (range 0.71–0.93). Ninety-five-percentile Hausdorff distance was computed with open-source software for image computation (Plastimatch^14^ v. 1.7.0), yielding 6.4 ± 3.6 mm (range 2.7–12.9 mm) for small bowel and 4.8 ± 2.4 mm (range 2.7–11.4 mm) for stomach/duodenum. The repeat segmentations from the two observers are available in RT structure sets associated with ten of the original-format CBCT image sets (described in the Data Records section).

## Usage Notes

There is some variability in image quality among the CBCT images in this dataset. The longer acquisition times (compared to diagnostic CT) rendered the images more prone to motion-induced artefacts, such as image blurring and streaking from implanted fiducials or stents. Since most CBCT scans were acquired with multiple breath-holds, there may be artefacts from variability in the breath-hold levels. Gas pockets in bowel and stomach were often present and contributed to streaking artefacts. CBCT scans of large and obese patients in some cases resulted in noisy images. Cupping artefacts, visible as a brightening near the perimeter of the axial-view images, were present in most of the limited-view CBCT reconstructions. These artefacts in some cases obscured the visibility of OAR boundaries and thereby increased the uncertainty in the manual segmentations in those areas.

During the image review process, we observed that the image quality of the datasets improved over time. In the earlier treatment cases, there was a learning curve for the technologists to become familiar with the imaging process, as well as coaching the patients to maintain a consistent breath hold. Therefore, the images showed increased likelihood for noise and residual motion artifacts. Only about one-quarter of the initial patient cases yielded at least two CBCT scans were usable for drawing contours. In later cases, technologists had gained experience with image acquisition and with coaching patient breath-hold. We found that about one-half of the latter patient cases yielded usable CBCT scans. As mentioned above, the obscured visibility of OAR boundaries in lesser quality images increases the uncertainty in manual segmentations, leading to larger variability between different observers. We would therefore expect a correspondingly larger variability in the predictions of the registration/auto-segmentation algorithm relative to manual segmentations used as a reference for evaluation.

As mentioned previously, the majority of the CBCT images are limited-view scans and the remainder are full-view scans. The presence of cupping artefacts in the limited-view CBCT scans may affect image intensity-based processing differently than for full-view CBCT. As stated previously, most of the manual OAR segmentations were limited to within the VOI on the CBCT images, and within an approximately 2 cm expansion of the VOI on the planning CT. It is important to note that, although segmentations may be present outside these limits in some cases, only the segmentations within the limits were reviewed and edited for accuracy (as described in the Methods section).

For each patient, the planning CT and two CBCT scans have been processed to have the same reconstructed dimensions, but the image dimensions across patients are different; therefore, if an algorithm requires one reconstructed dimension, researchers should further resample all the images and structure files.

Researchers may find this dataset useful for several applications. The images from patients treated for LAPC can serve to test the performance of multi-modality deformable CT-to-CBCT registration or auto-segmentation algorithms. The physician-drawn OAR segmentations provide a criterion standard for developing and training the algorithms, or can be used for validation. Researchers can collaborate with radiation oncologists to delineate additional normal or tumour-bearing tissues of interest, thereby expanding number of structures for training and validation. Finally, the dataset can serve to evaluate and commission commercially available registration and auto-segmentation capabilities. This is also particularly interesting as a stepping stone towards developing a cumulative dose-volume histogram for mobile structures such as the gastrointestinal tract that would be very helpful in determining the safety of dose escalation strategies^4^.

## Data Availability

Most of the processing of image data, including image registration, segmentation and technical validation (i.e., calculation of Dice similarity coefficients) was accomplished using commercially available software (MIM v. 6.9.7, MIM Software Inc.). Calculation of 95-percentile Hausdorff distance for technical validation used open-source software for image computation (Plastimatch^14^ v. 1.7.0).
